# A portable electrochemiluminescence aptasensor for β-lactoglobulin detection

**DOI:** 10.1007/s00216-022-04328-5

**Published:** 2022-09-21

**Authors:** Rossella Svigelj, Ivan Zuliani, Nicolò Dossi, Rosanna Toniolo

**Affiliations:** Department of Agrifood, Environmental and Animal Science, University of Udine, via Cotonificio 108, 33100 Udine, Italy

**Keywords:** Aptasensor, Biosensor, ECL, β-Lactoglobulin, Food analysis

## Abstract

Cow’s milk allergy is one of the most common food allergies in children with a prevalence of around 2.5%. Milk contains several allergens; the main ones are caseins and β-lactoglobulin (β-LG). At regulatory level, β-LG is not explicitly named, but milk is included in the list of substances or products causing allergies or intolerances. Hence, the presence of β-LG can be a useful marker for determining the presence of milk in food. In this work, we present an aptasensor based on electrochemiluminescence (ECL) for the quantification of β-LG in real food matrices displaying integrated advantages consisting of high specificity, good sensitivity, portability, and cost effectiveness. The performance and applicability of this sensor were tested by analyzing a sample of skimmed milk and an oat-based drink proposed as a vegetable substitute for milk of animal origin. We obtained a linear correlation between the intensity of the signal and the concentration of β-LG standard solutions (*y* = *x* * 0.00653 + 1.038, *R*^2^ = 0.99). The limit of detection (LOD) and the limit of quantification (LOQ) were found to be 1.36 and 4.55 μg L^−1^, respectively.

**Graphical abstract:**

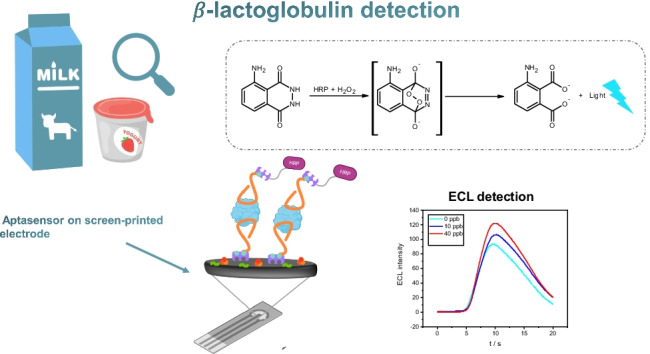

**Supplementary Information:**

The online version contains supplementary material available at 10.1007/s00216-022-04328-5.

## Introduction

Food allergens can cause abnormal reactions of the immune system in hypersensitive subjects [1]. The type and intensity of symptoms include swelling, asthma, abdominal pain, skin rashes, diarrhea, and in severe cases, anaphylactic shock [2, 3].

About 90% of food allergies are linked to allergens contained in milk, eggs, fish, shellfish, tree nuts, peanuts, soy, and wheat, also referred to as Big-8 [4, 5]. In Europe, producers are obliged to declare in the list of ingredients the presence of products considered food allergens [6]. In the absence of a list of ingredients, the presence of the allergen must be indicated on the package with the words “contains” followed by the name of the allergen. The risk of consuming food containing allergens not declared on the label is mainly attributable to cross-contamination [7]. This problem can be minimized by applying good hygiene practices (GHPs) and good manufacturing practices (GMPs) [8]. In cases where it is not possible, food companies should report precautionary terms such as “may contain” or “may contain traces of” relating to the allergen in question [9–11].

Cow’s milk allergy is one of the most common food allergies in children with a prevalence of around 2.5% [12]. Milk contains several allergens; the main ones are caseins and β-Lactoglobulin (β-LG) [13]. β-LG is a protein relatively resistant to acid hydrolysis and protease activity [14]. These characteristics allow it to preserve a certain structural integrity after digestion and to arrive intact in the intestinal mucosa where it elicits the immune response [15].

At regulatory level, β-LG is not explicitly named. However, since milk is included in the list of 14 allergens present in Annex II of regulation 1169/2011 [16], the presence of β-LG can be a useful marker for determining the presence of milk in food.

Immunochemical techniques were among the first techniques developed for the determination of β-LG in food and are still among the most used techniques for the determination of this protein [17, 18]. Also, several biosensors have been proposed for its detection based both on antibodies [19] and aptamers [20–22]. Obtaining highly sensitive and effective sensors is crucial in the determination of allergens, since their presence in unlabelled products could reach levels that are of public health relevance [23]. In this context, aptamer-based biosensors have greatly contributed to improve the quantification of allergens [24–28], allowing the diffusion of simpler, cheaper, and portable approaches.

In this work, we present a portable platform based on electrochemiluminescence (ECL) for the quantification of β-LG in real food matrices. The proposed strategy employs luminol (5-amino-2,3-dihydro-1,4-phthalazindione) which is a widely used emitter in ECL [29]. ECL has several advantages over the use of single techniques such as chemiluminescence, photoluminescence, and electroanalytical techniques [30–33]. The electrochemical reaction allows the control of the light emission reaction through the applied potential. Moreover, the ECL is more selective than chemiluminescence, since the generation of excited states in ECL can be selectively controlled by modifying the potentials applied to the electrode [34].

Here, an aptamer-based sandwich-type assay was developed on carbon screen printed using a miniaturized instrumentation [35]. The performance and applicability of the sensor were tested by analyzing a sample of skimmed milk and an oat-based drink proposed as a vegetable substitute for milk of animal origin.

## Materials and methods

### Chemicals and reagents

5′-tagged (biotin) BLG14 aptamer (sequence: GAC GAT CGG ACC GCA GTA CCC ACC CAC CAG CCC CAA CAT CAT GCC CAT CCG TGT GTG) was obtained from HPLC-purified from Sigma Aldrich (Italy), and the secondary structure is reported in Figure S1. Bovine serum albumin (BSA), sorbitol, biotin, mercaptoundodecanoic acid (MUA), mercaptohexanol (MH), sodium acetate, N-hydroxysuccinimide (NHS), 1-ethyl-3-[3-dimethylaminopropyl] carbodiimide hydrochloride (EDC), β-lactoglobulin, ethanolamine, 3,3′,5,5′-tetramethylbenzidine (TMB), and luminol were purchased from Sigma Aldrich (Italy). Streptavidin and horseradish peroxidase (HRP) conjugated with streptavidin were purchased from Merck (Italy). Ultrapure water (*R* > 18 MΩ) was obtained by means of an Elga Purelab flex 4 system (Veolia Water Technologies, Italy) and used for the preparation of buffer solutions.

### Apparatus and electrochemical measurements

Screen-printed carbon and gold electrodes were purchased by Dropsens (Metrohm, Italy). All chronoamperometric, impedimetric, and voltammetric measurements were performed using an Autolab PGSTAT204 potentiostat (Metrohm, Italy) managed by Nova software.

Electrochemiluminescence (ECL) measurements were performed by a µStat ECL portable bipotentiostat/galvanostat combined with a Si-photodiode integrated in the ECL cell (DropSens Metrohm, Italy) (see Figure S2) and controlled by DropView 8400 software.

The electrochemical impedance spectroscopy measurements were conducted using the redox probe [Fe(CN)_6_]^4−^/[Fe(CN)_6_]^3−^ 2 mM each, and KCl 3 mM in PBS. The applied potential was 0.115 V (half-wave potential of the redox pair), while the frequency was varied in the range from 10.000 to 0.01 Hz, with an amplitude of 0.005 V. The resistance to charge transfer (*R*_ct_) was calculated using NOVA software.

In the ECL measurements, the initial potential was equal to 0.1 V, the final potential was equal to 1.1 V, and the scanning speed was equal to 0.05 V/s.

### Aptamer binding affinity

The gold working electrode (WE) was activated electrochemically by cyclic voltammetry in 40 µL of 0.5 M H_2_SO_4_ (initial potential was equal to 0 V, inversion potential was equal to 1.3 V, scan rate was equal to 100 mV/s, number of cycles was equal to 10). The gold screen-printed electrode (AuSPE) was then washed with ethanol and dried with air. Subsequently, the surface of the AuSPE was covered with 10 µL of MH and MUA 3:1 10 µM each in 10 mM acetate buffer at pH 5.5 overnight. Then the electrode was covered with 20 µL of 0.4 M EDC and 0.2 M NHS solution in acetate buffer pH 5.5 for 30 min. Next, the electrode surface was incubated with 20 µL of the solution containing 50 ppm of β-LG in acetate buffer pH 5.5 for 30 min. Finally, the WE of the AuSPE was washed and covered with 20 µL of 1 M ethanolamine in PBS pH 7.4, incubated for 15 min, and washed with three 250 µL aliquots of selection buffer (BS-LG; 50 mM Tris pH 7.4, 150 mM NaCl, 2 mM MgCl_2_).

Twenty µL of different concentrations of BLG14 aptamer (40, 60, 80, and 100 nM) were deposited on the WE of the AuSPE. After half an hour of incubation, the surface of the electrode was washed with BS-LG and covered with 20 µL of the 0.75 µg/ml streptavidin-HRP solution in BS-LG, for 10 min. Lastly, 40 µL of the TMB substrate were deposited on the AuSPE. After 60 s of incubation, a chronoamperometric measurement was carried out at 0 V for 50 s. A schematic representation of the procedure is shown in Figure S3.

### Sandwich assay on screen-printed carbon electrodes and electrochemiluminescence (ECL) measurements

The surface of the working electrode (WE) of the SPCE was washed with 500 µL of ethanol and dried with air. Subsequently, the WE was covered with 10 µL of streptavidin 1 mg/ml and incubated overnight. Then a blocking step was performed with 20 µL of 1% BSA-6% sorbitol in PBS pH 7.4 for 30 min. Subsequently, 20 µL of BLG14-biotin aptamer 1 µM in PBS pH 7.4 was incubated for 30 min. Finally, the WE of the SPCE was covered with 20 µL of 0.5 µM biotin in PBS pH 7.4, for 10 min.

**Fig. 1:**
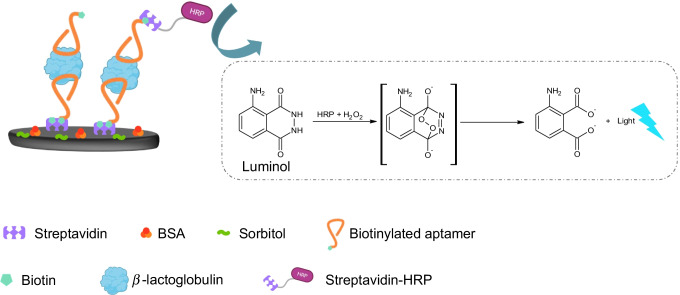
Schematic representation of the sandwich aptasensor design and working principle. As can be seen, the aptamer was anchored through biotin–streptavidin interaction; non-specific interactions were avoided thanks to a blocking step with BSA and sorbitol; then after the interaction with β-LG, the detection aptamer was incubated on the electrode; and after labeling with streptavidin-HRP conjugate, it was possible to perform the measurement of luminol substrate

Twenty µL of the solutions at different concentrations of β-LG were deposited on the WE of the SPCE. After 30 min of incubation with standard solutions, the surface of the WE of the SPCE was washed. Subsequently, 20 µL of the biotinylated aptamer was deposited on the WE surface of the SPCE to complete the aptamer-β-LG-aptamer sandwich. After 30 min of incubation, the WE of the SPCE was washed with three 250 µL aliquots of buffer, dried with air, and covered with 20 µL of a streptavidin-HRP solution for 10 min. Subsequently, 40 µL of luminol 2 mM and H_2_O_2_ 10 µM probe in Tris pH 9 were deposited in such a way as to cover all three electrodes. Then, after 60 s of incubation, the measurement in ECL was carried out. The initial potential was equal to 0.1 V, the final potential was equal to 1.1 V, and the scanning speed was equal to 0.05 V/s. Figure 1 shows a schematic representation of the biosensor assembly and working principle.

### Real sample analysis

A sample of skimmed cow’s milk and a sample of an oat-based drink, proposed as a vegetable substitute for milk of animal origin, were analyzed. The cow’s milk was diluted to bring the β-LG content in the sample within the linear response range. The oat-based drink was instead subjected to centrifugation at 3500 rpm for 30 min, and the supernatant was diluted 1/20 to reduce the matrix effect. Both samples were spiked with 5 μg L^−1^ and of β-LG.

## Results and discussion

### Evaluation of aptamer affinity against β-lactoglobulin

**Fig. 2:**
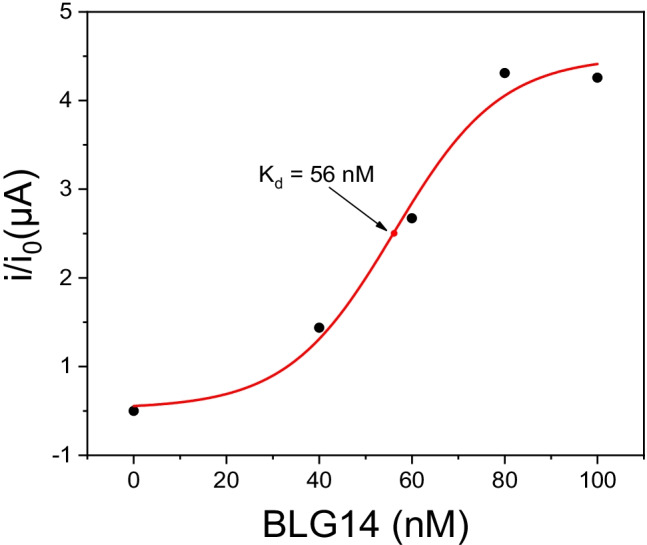
Binding curve of BLG14 aptamer against β-LG

To determine the affinity of the aptamer for β-LG, the dissociation constant (*K*_d_) of the β-lactoglobulin-aptamer adduct was determined using the procedure previously described. From the results obtained, shown in Fig. 2, the *K*_d_ of the β-lactoglobulin-BLG14 adduct, identifiable as the inflection point in the titration curve, is 56 nM. This value is very similar to that previously reported in the literature during the SELEX procedure [20] and confirms the strong affinity of the aptamer for β-LG.

### Electrochemical impedance spectroscopy (EIS) characterization of the biosensor

**Fig. 3:**
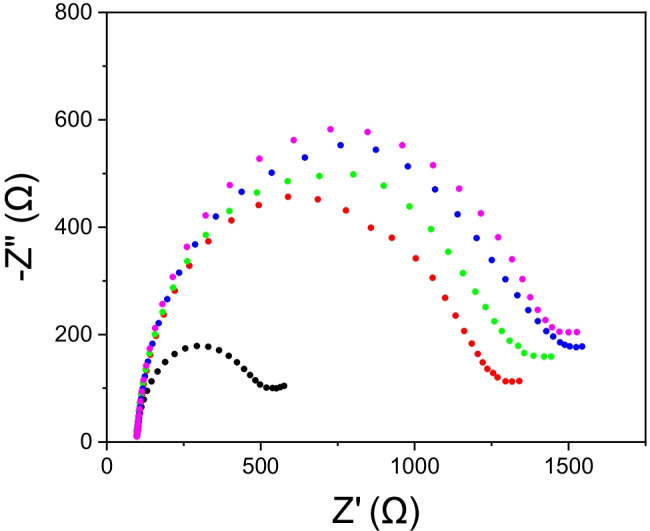
Nyquist plots recorded on SPCE using the redox probe [Fe(CN)_6_]^4−^/[Fe(CN)_6_]^3−^ 2 mM each, KCl 3 mM in PBS: bare electrode (black, R_ct_ = 429 Ω), modified with streptavidin (red, R_ct_ = 1117 Ω), after 30 min of BSA-sorbitol (green, R_ct_ = 1214 Ω), after 30 min of BLG14 (blue, R_ct_ = 1320 Ω), after 10 min of biotin (pink, R_ct_ = 1354 Ω)

To verify the effectiveness of the SPCE modification procedure, a surface characterization was performed at the end of each modification step using EIS. The results are shown in Fig. 3. The Nyquist diagram confirms the correct SPCE surface modification strategy. From the Nyquist plots, it is possible to observe how the impedance increases following each modification step. This is attributable to an increase in the material deposited on the electrode surface following each phase and is therefore related to the correct functioning of the modification procedure.

### Lactoglobulin quantification by ECL aptasensor

This sensor uses a sandwich-type approach, in which the detection aptamer is labeled with biotin, where the streptavidin-HRP conjugate will bind in proportion to the presence of β-LG. Luminol is widely used in ECL and has an emission λ at 420 nm. The reaction can be catalyzed by the presence of different oxidases such as HRP. Usually, the coreagent associated with luminol is hydrogen peroxide. The reaction requires an alkaline pH, and the application of a potential is equal to or greater than + 0.5 V. In our case, we performed a linear sweep voltammetry from 0.1 to 1.1 V in the presence of luminol, H_2_O_2_, and HRP. The electrochemiluminescent and electrochemical signals were simultaneously generated and recorded. We initially conducted a study on the behavior of solutions containing only luminol, luminol, and H_2_O_2_ and finally luminol H_2_O_2_ and HRP. The results are shown in Figure S4 and show how the ECL emission intensity of the luminol alone is low while it increases with the presence of hydrogen peroxide and again with HRP. In detail, in Figure S4A, the electrochemical response shows an oxidation peak around + 0.35 V associated with the luminol oxidation. In Figure S4B, ECL signal of luminol in the presence of hydrogen peroxide and HRP (green line) shows its characteristic behavior, and the emission of light increases during the oxidation of luminol. ECL and electrochemical peaks match around + 0.35 V.

**Fig. 4:**
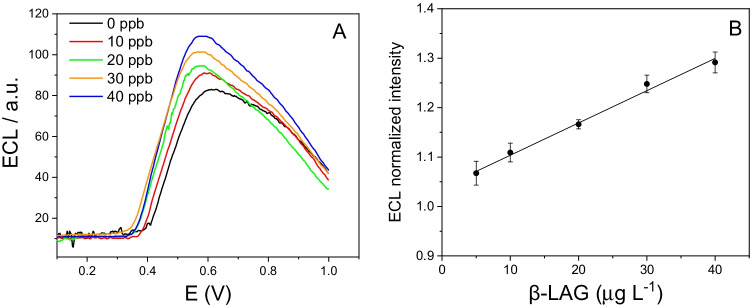
ECL measurements obtained for solutions at concentrations between 0 and 40 µg L^−1^ of β-LG (**A**) and corresponding calibration line (**B**)

For the quantification of β-LG, we therefore exploited this increase in emission in the presence of HRP. We considered the maximum of electrochemiluminescence emission for quantification (see Fig. 4A). In this case, the potential at which we recorded the maximum signal is shifted to more positive potentials than those recorded on bare electrode (Figure S4B) as expected for a modified electrode. The signals obtained for the standard solutions of β-LG were evaluated as a ratio with the signal recorded on the blank (0 µg L^−1^). Figure 4B clearly shows a linear correlation between the intensity of the signal and the concentration of β-LG in the analyzed solution (*y* = *x* * 0.00653 + 1.038, *R*^2^ = 0.99).

The limit of detection (LOD), calculated as three times the standard deviation of the blank signal divided by the slope calculated for the linear dynamic range, and the limit of quantification (LOQ), estimated by multiplying the LOD by 3.3, were found to be 1.36 and 4.55 μg L^−1^, respectively, while the inter-assay % CV was 1.5; all these parameters are summarized in Table 1. The limit of detection of the ECL aptasensor successfully competes with that of the immunoassays present on the market, as it is evident from the data summarized in Table 2, which compiles a variety of commercial tests using different approaches with the related LOD.

**Table 1:** Performance of the ECL aptasensor

Parameter	Performance
*R*^2^	0.99
Linear range (µg L^−1^)	5–40
LOD (µg L^−1^)	1.36
LOQ (µg L^−1^)	4.55
Inter-assay % CV	1.5

**Table 2:** Comparison of the analytical performance of commercial ELISA assays for β-LG

Manufacturer	ELISA kit	Principle	LOD
Cusabio	Bovine Beta-Lactoglobulin ELISA Kit	Competitive	0.12 μg mL^−1^
LSBio	Bovine Beta-Lactoglobulin ELISA Kit	Sandwich	1.56 μg mL^−1^
RIDASCREEN	RIDASCREEN FAST β-Lactoglobulin	Sandwich	0.16 mg kg^−1^
RIDASCREEN	RIDASCREEN β-Lactoglobulin	Competitive	1.4 mg kg^−1^
Eurofins	SENSISpec ELISA Beta-Lactoglobulin	Sandwich	1.5 μg kg^−1^
Oxoid	ELISA Systems Beta-Lactoglobulin Residue Detection Kit for Food Allergen Testing	Sandwich	0.1 μg mL^−1^

### Real sample analysis

A sample of skimmed cow’s milk and a sample of an oat-based drink proposed as a vegetable substitute for milk of animal origin were analyzed. Table 3 shows for each sample the addition of the experimental β-LG content, the theoretical concentration of the added diluted samples, and the recovery. The theoretical concentration of the added samples takes into account the initial concentration of the sample determined experimentally, the addition of β-LG, and the dilution attributable to the addition itself.

**Table 3:** β-LG quantification in different food matrices with the ECL aptasensor.

Sample	β-LG concentration (μg L^−1^)* (ppb)^*^	β-LG concentration (μg L^−1^)**	Recovery (%) ***
Skimmed milk	10–20	17.08 ± 2.47	\
Skimmed milk spiked with 5 ppb	20.37	19.97 ± 2.04	98.01
Oat milk	0	< LOD	\
Oat milk spiked with 5 ppb	5	4.74 ± 1.13	94.71

*The expected concentration in the added samples was calculated considering the initial concentration, the addition of β-LG, and the dilution attributable to the addition itself.

**Average of three repetitions.

***Obtained as the ratio between β-LG concentration determined and that expected in the spiked samples.

**Fig. 5:**
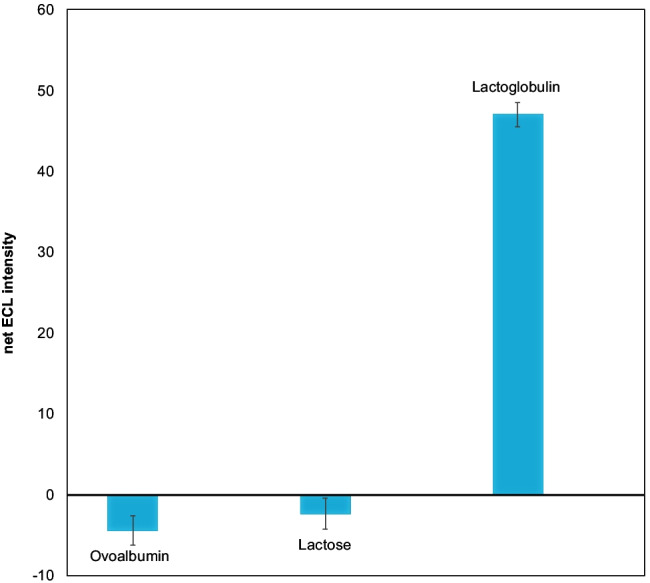
Selectivity tests: the sensor was tested with 40 μg L^−1^ of ovoalbumin, 40 μg L^−1^ of lactose and 40 μg L^−1^of β-LG

The recoveries calculated for the spiked samples are close to 100% confirming the aptasensor’s ability to provide accurate and reproducible data in these matrices. The selectivity test against lactose, one of the major components of milk, and ovoalbumin, shown in Fig. 5, confirms the appropriateness of the method for the selective quantification of β-LG in real matrices.

## Conclusions

In this work, we have proposed a portable, low-cost, and easy-to-use biosensor. The ECL aptasensor developed was built on disposable graphite screen-printed electrodes, adopting a sandwich approach, in which the detection aptamer was labeled with biotin, on which the streptavidin-HRP conjugate bound in proportion to the presence of β-LG.

We obtained a linear correlation between the intensity of the signal and the concentration of β-LG standard solutions (*y* = *x* * 0.00653 + 1.038, *R*^2^ = 0.99). The limit of detection (LOD) and the limit of quantification (LOQ) were found to be 1.36 and 4.55 μg L^−1^of β-LG. The inter-assay % CV was 1.5. Thus, the performance of the proposed method, which covers a linear response in a wide concentration range, makes it an effective tool in food quality control and cross-contamination monitoring.

## Supplementary Information

Below is the link to the electronic supplementary material.

Supplementary file1 (DOCX 1270 KB)

## Data Availability

Not applicable.
