# Supplementary material for: A portable electrochemiluminescence aptasensor for β-lactoglobulin detection
Source: Anal Bioanal Chem. 2022 Sep 21;414(27):7935–41. doi: 10.1007/s00216-022-04328-5 (PMC9568494; doi:10.1007/s00216-022-04328-5)
Supplement: Supplementary file 1 — Supplementary file1 (DOCX 1270 KB) [file 216_2022_4328_MOESM1_ESM.docx]

**A portable electrochemiluminescence aptasensor** **for ß-lactoglobulin detection**

Rossella Svigelj^1,*^, Ivan Zuliani^1^, Nicolò Dossi^1^, Rosanna Toniolo^1,*^

^1^Department of Agrifood, Environmental and Animal Science, University of Udine, via Cotonificio 108, 33100, Udine, Italy;

**^*^Corresponding authors:**

Rossella Svigelj (rossella.svigelj@uniud.it)

Rosanna Toniolo (rosanna.toniolo@uniud.it)


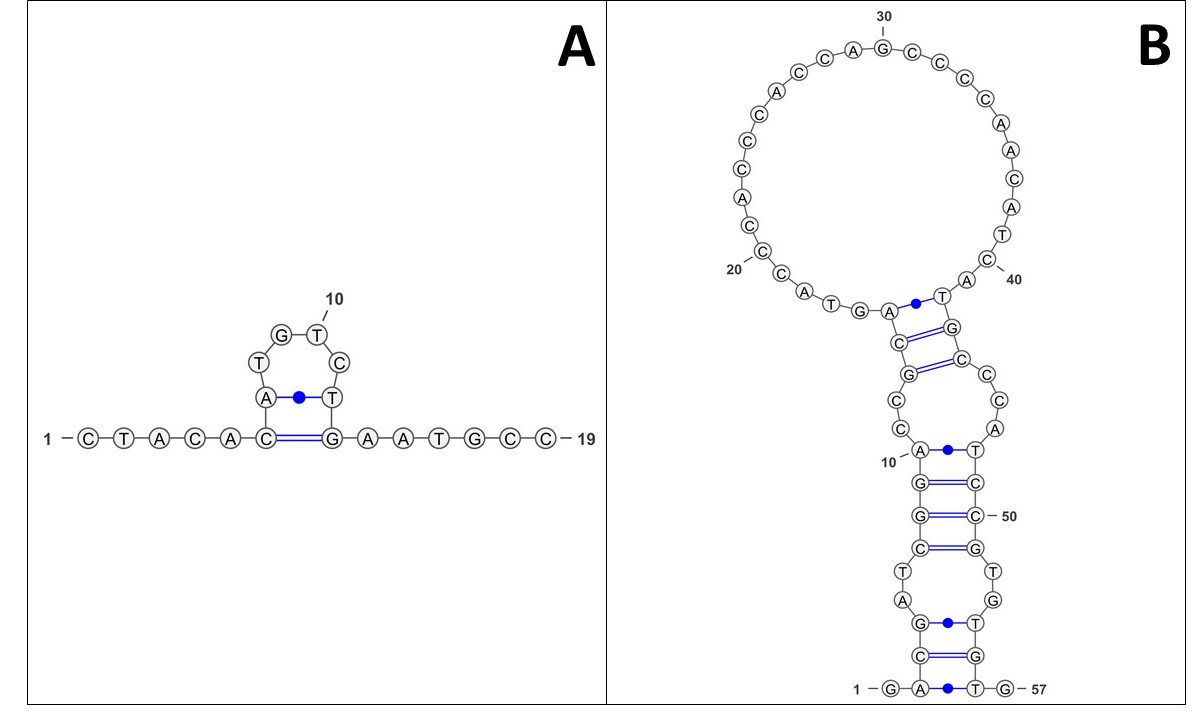


ΔG (kcal/mol) = −2.52

**Figure S1** Secondary structure of BLG14.

**Figure S2** µStat-ECL: the electrochemiluminescence (ECL) cell that is connected to the potentiostat, controlled by a specific software that allows recording simultaneously electrochemical and ECL data.

**Figure S3** Schematic representation of the various modification steps of the SPAuE surface aimed at determining the K_d_ of the BLG14 aptamer.

**Figure S4** (A) Cyclic voltammograms of luminol (black), luminol in presence of H_2_O_2_ (red) and luminol in presence of H_2_O_2_ and HRP (green). (B) ECL response for luminol (black), luminol in presence of H_2_O_2_ (red) and luminol in presence of H_2_O_2_ and HRP (green).
